# Characteristics and outcomes of gallbladder cancer patients at the Tata Medical Center, Kolkata 2017–2019

**DOI:** 10.1002/cam4.5677

**Published:** 2023-02-13

**Authors:** Anindita Dutta, Tushar Mungle, Nandita Chowdhury, Pritha Banerjee, Anisha Gehani, Saugata Sen, Mohandas Mallath, Paromita Roy, Shekhar Krishnan, Sandip Ganguly, Sudeep Banerjee, Manas Roy, Vaskar Saha

**Affiliations:** ^1^ Cell Biology Tata Translational Cancer Research Centre Kolkata India; ^2^ Division of Cancer Sciences University of Manchester Manchester UK; ^3^ Clinical Research Unit Tata Translational Cancer Research Centre Kolkata India; ^4^ Department of Radiology Tata Medical Center Kolkata India; ^5^ Department of Digestive Diseases Tata Medical Center Kolkata India; ^6^ Department of Histopathology Tata Medical Center Kolkata India; ^7^ Department of Paediatric Haematology and Oncology Tata Medical Center Kolkata India; ^8^ Department of Medical Oncology Tata Medical Center Kolkata India; ^9^ Department of Gastrointestinal and Hepatobiliary Surgery Tata Medical Center Kolkata India

**Keywords:** clinicopathology, electronic medical records, gallbladder cancer, treatment

## Abstract

**Background:**

The north and north‐eastern regions of India have among the highest incidence of gallbladder cancer (GBC) in the world. We report the clinicopathological charateristics and outcome of GBC patients in India.

**Methods:**

Electronic medical records of patients diagnosed with GBC at Tata Medical Center, Kolkata between 2017 and 2019 were analyzed.

**Results:**

There were 698 cases of confirmed GBC with a median age of 58 (IQR: 50–65) years and female:male ratio of 1.96. At presentation, 91% (496/544) had stage III/IV disease and 30% (189/640) had incidental GBC. The 2‐year overall survival (OS) was 100% (95% CI: 100–100); 61% (95% CI: 45–83); 30% (95% CI: 21–43); and 9% (95% CI: 6–13) for stages I–IV, respectively (*p* = <0.0001).   For all patients, the 2‐year OS in patients who had a radical cholecystectomy followed by adjuvant therapy (*N* = 36) was 50% (95% CI: 39–64), compared to 29% (95% CI: 22–38) for those who had a simple cholecystectomy and/or chemotherapy (*N* = 265) and 9% (95% CI: 6–14) in patients who were palliated (*N* = 107) (*p* = <0.0001).

**Conclusion:**

The combined surgical/chemotherapy approach for patients with stage II GBC showed the best outcomes. Early detection of GBC remains problematic with the majority of patients presenting with stage III–IV and who have a median survival of 9.1 months. Our data suggests that the tumor is chemoresponsive and multi‐center collaborative clinical trials to identify alternative therapies are urgently required.

## Graphical abstract

The curative approach for gallbladder cancer (GBC) is radical cholecystectomy with adjuvant chemotherapy. GBC has non‐specific symptoms, so screening/early detection is not possible. We report that 90% of GBC patients presented with late‐stage inoperable disease. Patients with late‐stage disease who received chemotherapy had significantly better survival over those who did not (*p* < 0.0001). Our real‐world data suggests that GBC is a chemosensitive disease. Clinical trials in low‐middle income countries to reposition available therapies are urgently required.

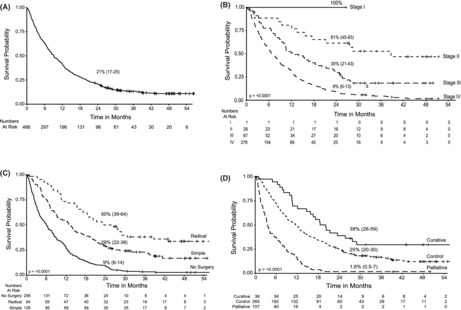

## 1 INTRODUCTION

Gallbladder cancer (GBC) is the sixth commonest of gastrointestinal cancers, with wide geographic variations in prevalence.^1^ India has one of the highest incidences worldwide, with the disease occurring primarily in the north‐east and eastern regions of the country.^2^, ^3^, ^4^ Though etiology remains unknown, evidence suggests that GBC arises from chronic inflammation progressing to dysplasia, carcinoma‐in‐situ, and finally invasive cancer.^3^ GBC is more common in females and occurs at a younger age in India.^5^ Symptoms are insidious with around 30% of GBCs diagnosed incidentally (IGBC) at the time of laparoscopic cholecystectomy.^6^, ^25^ Majority of symptomatic GBC present with jaundice. Pre‐operatively GBC is suspected in only 30% of patients, where the disease is either locally advanced or obstructing biliary outflow and often presents with nodal involvement or distant metastases.^7^ In around 10% of patients, disease is localized to the gallbladder. In these patients, radical cholecystectomy with adjuvant therapy is the standard‐of‐care^8^, ^9^ and can achieve 5‐year survival rates of 63%.^7^, ^10^

Tata Medical Centre (TMC) is a charitable not‐for‐profit tertiary cancer care center established in 2011 in Kolkata with a catchment area covering Eastern, North, and North‐eastern India. The hospital benefits from electronic patient records, state‐of‐the‐art diagnostic facilities, and a multidisciplinary approach to GBC. Here we report our experience with patients diagnosed with GBC between 2017 and 2019 as a retrospective cohort study.

## 2 PATIENTS AND METHODS

TMC is paperless with an electronic medical records (EMR) system which contains all necessary clinical information. Data in the EMR is recorded and stored in an Oracle database. MySQL was used to extract stored information at time of analysis. Institutional Review Board (IRB) approval was obtained for this study, as a retrospective analysis of pseudo‐anonymized data with no impact for patient outcomes, consent was not required.

### 2.1 Patient identification and data extraction

The EMR was interrogated to identify patients who had “gallbladder” reported in the text of the diagnostic assessment module between January 1, 2017 and December 31, 2019, excluding those with a diagnosis of benign cholecystitis. Data extracted from the EMR were manually inspected to confirm a diagnosis of GBC. Patients in whom either radiological or pathological diagnosis of GBC could not be confirmed were excluded from further analyses. Data extracted from EMR is available as fixed text forms where uniformity is preserved and were categorized as structured. Other fields allow free text and were categorized as unstructured. Structured data included patient demographics, body mass index (BMI) at the timepoint of first visit, and results of laboratory investigations. Unstructured data including information on comorbidities, stage, radiology, pathology, treatment schedules, and outcomes. These were independently interpreted by at least two independent team members and verified where required by the clinician, radiologist, or pathologist.

### 2.2 Patient management

Patients presented to TMC with a history of jaundice or suspected metastatic disease or a simple cholecystectomy elsewhere with a pathological diagnosis of IGBC. Diagnosis of GBC was confirmed by pathological examination of biopsy specimens. Computerized tomography (CT) scans were used to establish the extent of disease. For localized disease, surgery followed by adjuvant therapy was offered as treatment. Patients who had unresectable disease were offered palliative therapy or palliation based on their general condition. (Figure S1).

### 2.3 Study definitions

For patients with IGBC, the date of simple cholecystectomy was taken as the date of diagnosis. For other patients the date of confirmatory radiology or pathology was used as the date of diagnosis. Disease was staged with the TNM classification and graded based on the state of differentiation (American Joint Committee of Cancer).^12^ Categorization of pathological types and metastatic sites are described in Table S1A,B. Based on the CT scan report, tumors where the mass had spread beyond the gallbladder (GB) but remained confined to the locoregional area were classified as locally advanced GBC. Tumors originating from GB with spread to distant organs were classified as metastatic GBC. Where pathological TNM was not available, the presentation CT report was used for staging using the RECIST 1.1^13^ TNM classification.

It was not possible to accurately define the stage of disease at first presentation in patients who had prior radical resection elsewhere and then subsequently presented to TMC with more advanced disease. Therefore, for the purposes of this analyses, patients were grouped as follows: those with a radical cholecystectomy, with or without major liver resection, followed by adjuvant chemotherapy in Group 1; those in whom tumors burden negated surgery but who received chemotherapy/radiotherapy in Group 2, and those in whom only symptomatic management was possible were in Group 3. Treatment responses for patients who received at least three cycles of chemotherapy were assessed through CT‐guided imaging and compared with the initial TNM staging (RECIST 1.1 criteria) where possible. Where no antecedent history of type II diabetes was available, a diagnosis of co‐existing diabetes was based on the HbA1c test result. Biochemical laboratory test data were extracted to evaluate liver function at presentation.

Families/patients were contacted to collect follow‐up data. The censor dates for patients who presented to the hospital in years 2017, 2018, and 2019 were September 30, 2021, December 31, 2021, and January 31, 2022, respectively. Survival analyses was performed only for patients in whom follow‐up data were available.

### 2.4 Statistical analysis

Treatment responses based on stage of disease were compared using the Kruskal–Wallis test (for groups) and pairwise comparison performed using the Wilcoxon test. Categorical variables are reported as *n* (%). Continuous variables are reported as median [inter quartile range (IQR)]. Survival analyses were carried out using the Kaplan–Meier and log‐rank test used for comparison of subgroups. Cox regression was performed to assess the effect of prognostic covariates on outcome. All statistical analyses were performed using R statistical software (version 3.6.1) and RStudio (version 1.2.5019). *p*‐values less than 0.05 were considered significant.

## 3 RESULTS

**FIGURE 1:**
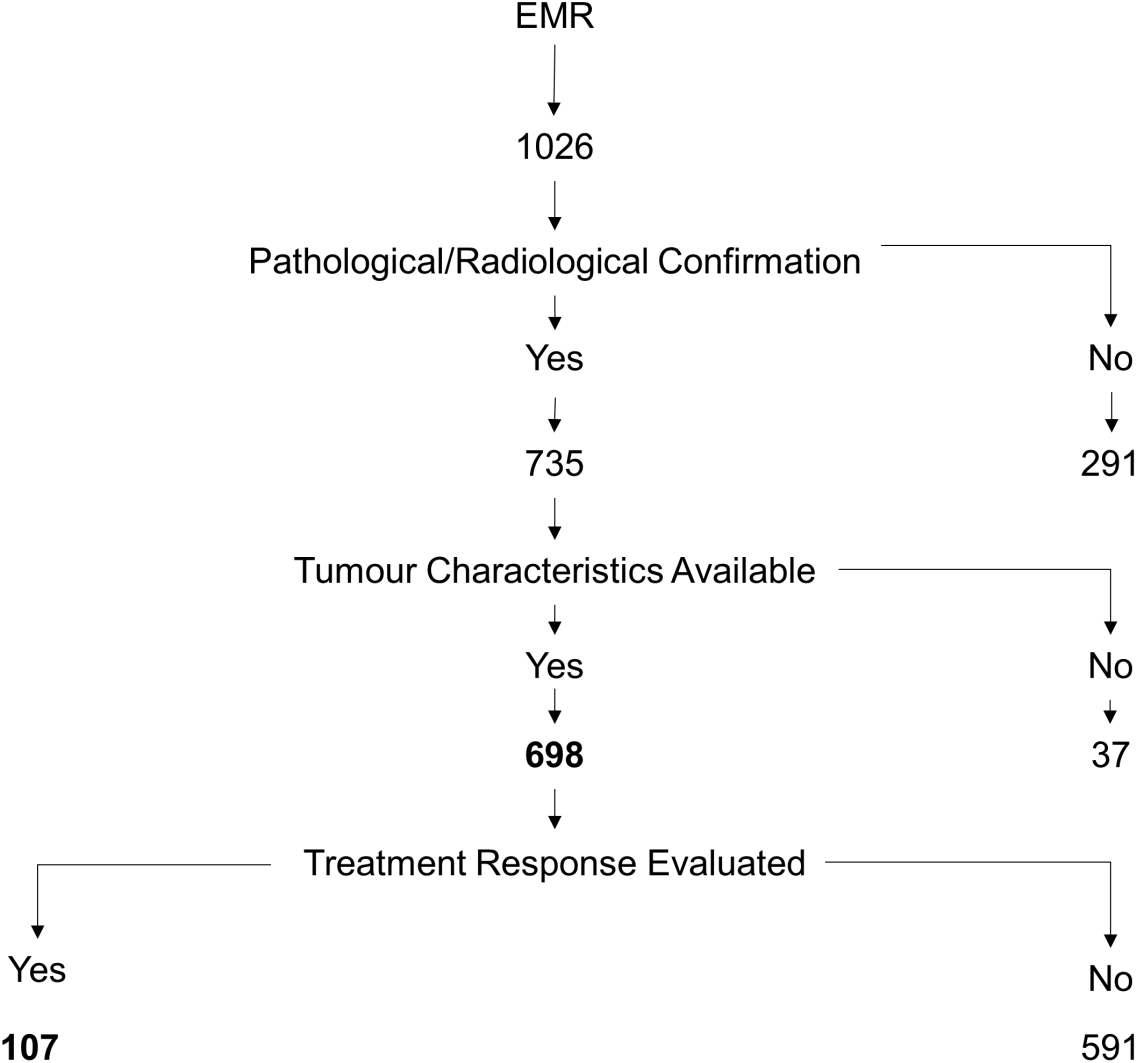
Identification of patients with gallbladder cancer from electronic medical records (EMR). Search term “gallbladder” excluding “benign cholecystitis”.

A total of 1,026 patients with disease site as gallbladder during the study period were identified. For 291 patients, a pathological or radiological confirmation of cancer were not available. Details on pathological subtypes were not available in 37. These patients were excluded from further analyses (Figure 1). The clinical and pathological details of 698 patients with GBC are presented in Table 1. The median age of the study cohort was 58 (IQR: 50–65 years) years with BMI of 22 (IQR: 19–25), and a female:male ratio of 1.96 (Table 1). Additional comorbidities were hypothyroidism in 11% (60/553), type II diabetes in 33% (183/560), and hypertension in 50% (237/473). A family history of cancer was reported in 19% (61/321), but further details were not available. At presentation to TMC, 67% (403/607) had an ECOG score of 0–1, gallstones were present in 57% (266/468), gallbladder polyps in eight patients, and 30% (189/640) were diagnosed with IGBC. In 10 of the IGBC patients, a diagnosis of GBC was missed on initial histopathological examination; of these five presented to TMC with port‐site metastases. Among the 55% (354/640) patients who had distant spread, more than half (59%) had liver metastases. Over 80% of patients were diagnosed with adenocarcinoma. About 14.5% (90/544) had TNM stage III disease while 75% (406/544) presented in stage IV. In 330 evaluable patients, 90% (296/330) patients had grade II/III adenocarcinoma. Elevated bilirubin and/or alkaline phosphatase levels suggestive of biliary tract obstruction were seen in 9% (38/422) of patients. Liver enzymes alone were elevated (≥3× normal) in 28% (120/422) while 18% (79/422) had essentially normal liver functions (Table 2 and Table S2).

**TABLE 1:** Characteristics of patients with gallbladder cancer diagnosed at Tata Medical Center, 2017–2019.

	Data available	*N* (%)
Patient characteristics	698	
Age		
Median [IQR]		58 [50–65]
Sex		
Male		236 (34)
Female		462 (66)
BMI		
Median [IQR]		22 [19–25]
Comorbidities		
Hypothyroidism	553	60 (11)
Diabetes	560	183 (33)
Hypertension	473	237 (50)
Family history of cancer	321	61 (19)
ECOG	607	
0		29 (5)
1		374 (62)
2		135 (22)
3		49 (8)
4		20 (3)
GallStones	468	
Yes		266 (57)
Polyp		8 (2)
Disease characteristics		
At presentation at TMC	640	
Incidental		189 (30)
Locally advanced		97 (15)
Metastatic		354 (55)
Metastasis sites	354	
Liver		225 (59)
Peritoneum & Omentum		49 (14)
Lungs		30 (8)
Others		43 (19)
Unknown		7 (55)
Pathology	698	
Adenocarcinoma		575 (82)
Adenosquamous carcinoma		23 (3)
Neuroendocrine carcinoma		12 (2)
Others		88 (13)
Stage	698	
0		1 (0.1)
I		4 (0.5)
II		43 (6.2)
III		90 (13)
IV		406 (58.2)
Unknown		154 (22)
AJCC grading	330	
1		33 (10)
2		145 (44)
3		152 (46)
Therapeutic strategy	642	
Palliative		169 (26)
Curative		57 (9)
Disease control		416 (65)

**TABLE 2:** Biochemical liver function test results at presentation

Bilirubin	SGOT	GGT	SGPT	AP	*N* (%)
High	Normal	Normal	Normal	–	21 (5)
High	Normal	Normal	Normal	High	17 (4)
High	±High	±High	±High	±High	134 (32)
Low	±High	±High	±High	±High	120 (28)

Of the 698 patients, 54 patients were not treated at TMC, and further details were not available. In total, 642 (92%) patients opted for treatment at TMC, and stage‐specific treatments offered are shown in Table 3. As shown in Figure S1 in stage II and III (when possible), a radical cholecystectomy with adjuvant chemotherapy is recommended. About 19 (44%)/43 with stage II disease, had a radical cholecystectomy of which 16 were performed at TMC and three done elsewhere. A total of 15/19 opted for adjuvant chemotherapy at TMC and four at hospitals closer to home. One patient opted for palliation and 23 (53%) patients who had simple cholecystectomy either refused further surgery or were deemed to be not suitable for a radical cholecystectomy. In stage III disease, 30 (36%)/84 (19 at TMC and 11 elsewhere) had a radical cholecystectomy. Twenty (24%) patients had a simple/partial cholecystectomy prior to coming to TMC. Thus 43 (34%)/127 patients with stage II/III disease had undergone simple cholecystectomy prior to referral in whom further surgery was not possible. In patients with stage IV disease, 20 (5%)/398 (11 at TMC, nine elsewhere) had a radical cholecystectomy. In total, 109 (28%) had a simple/partial cholecystectomy prior to coming to TMC of whom 92 (86%) had metastatic disease and 268 (67%) patients were offered disease control or palliation.

**TABLE 3:** Modalities of treatment of patients with GBC at Tata Medical Center, 2017–2019

Stage	Number	SCy	SCy + Ch/RT	Partial Cy	RCy	RCy + Ch/RT	Ch	PCa
0	1	1	–	–	–	–	–	–
1	4	2	1	–	1	–	–	–
2	43	18	5	–	4	15	–	1
3	84	14	5	1	10	20	22	12
4	398	50	57	2	6	15	135	133
Unknown	112	31	11	1	14	7	19	29
Total	642	116	79	4	35	57	176	175

Abbreviations: Ch, chemotherapy; Cy, cholecystectomy; PCa, palliative care; RCy, radical cholecystectomy; RT, radiotherapy; SCy, simple cholecystectomy.

**FIGURE 2:**
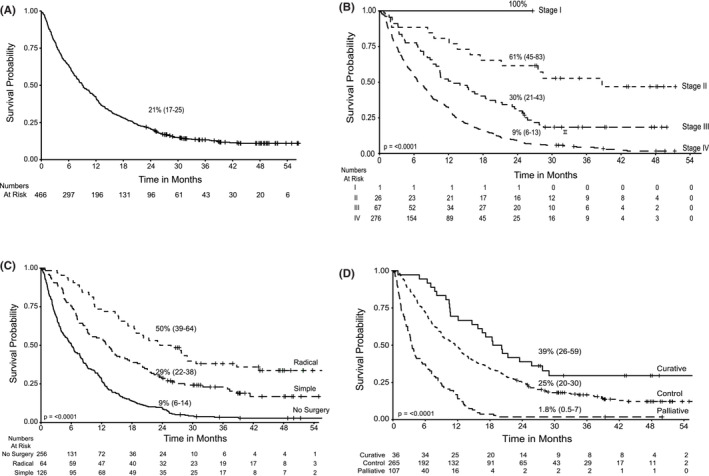
Kaplan–Meier survival analysis for (A) overall cohort (*n* = 466; median survival – 9.8 months); (B) stage I, II, III (median survival – 12.7 months), and IV (median survival – 6.8 months) sub‐cohorts; (C) sub‐cohorts who opted radical surgery (median survival – 24.6 months) and non‐radical surgery (median survival – 12.9 months); (D) sub‐cohorts, group 1 who opted for radical cholecystectomy along with chemotherapy (median survival – 19.4 months), group 2 who received sub‐optimal treatment that is, simple cholecystectomy along with chemotherapy or only chemotherapy (median survival – 11.5 months), and group 3 were palliative care group (median survival – 3.5 months).

Survival data was available for 478 patients. Twelve patients were excluded from the survival analysis as the date of event was unavailable. Fifty eight (12%) were alive at the time of analyses. Survival analysis with a median follow‐up time of ~2 years for 466 patients showed a 2‐year overall survival (OS) of 21% (95% CI: 17–25) with a median survival of 9.08 months (Figure 2A). The 2‐year OS was 100% (95% CI: 100–100); 61% (95% CI: 45–83); 30% (95% CI: 21–43); and 9% (95% CI: 6–13) for stages I, II, III, and IV, respectively (*p* = < 0.0001) (Figure 2B). Median survival was 38.7, 12.7, and 6.8 months for stages II, III, and IV, respectively. Patients with stage I disease had not reached median survival at the time of analysis. Of the 26 patients in stage II, a radical cholecystectomy was performed in 13 who had a 2‐year OS of 69% (95% CI: 37‐87) compared with 46% (95% CI: 19‐70) for 13 who had a simple cholecystectomy (*p* = 0.26). In stage III, outcomes were poor in patients who had no surgery and in stage IV, the few patients in whom a radical cholecystectomy could be performed had significantly better outcomes then those who had a simple cholecystectomy or no surgery (*p* = <0.0001) (Table S3).

Overall, the 2‐year OS for those undergoing radical cholecystectomy was 50% (95% CI: 39–64) with a median survival of 24.6 months compared to those with a prior simple cholecystectomy and no further surgery/adjuvant therapy where the 2‐year OS was 29% (95% CI: 22–38) with a median survival of 12.9 months (*p* = 0.0015). Patients who were not operated had a 2‐year OS of 9% (95% CI: 6–14) with a median survival of 6 months (Figure 2C). Group 1 patients receiving radical cholecystectomy followed by chemotherapy treatment had a 2‐year OS of 39% (95% CI: 26–59) and median survival of 19.4 months compared to 25% (95% CI: 20–30) with 11.5 months median survival for those in Group 2 with disease control receiving simple cholecystectomy followed by chemotherapy or chemotherapy alone. Patients in Group 3 (treated with palliative intent) had a median survival of 3.5 months, OS of 1.8% (95% CI: 0.5–7) (*p* = <0.0001) (Figure 2D). A multivariate cox regression analysis was performed to assess effect of covariates on time to event (death). A cohort of 466 patients with survival data was included with analysis carried out on 123 patients with events for 102 patients (rest omitted due to missing values). The *p*‐value for all three overall tests (likelihood, Wald, and score) from the cox proportion model was significant (*p*‐value <0.001) with the only parameter ECOG (value = 3) (*p* = 0.036) having strong relationship with increased risk of death (Table S4).

**FIGURE 3:**
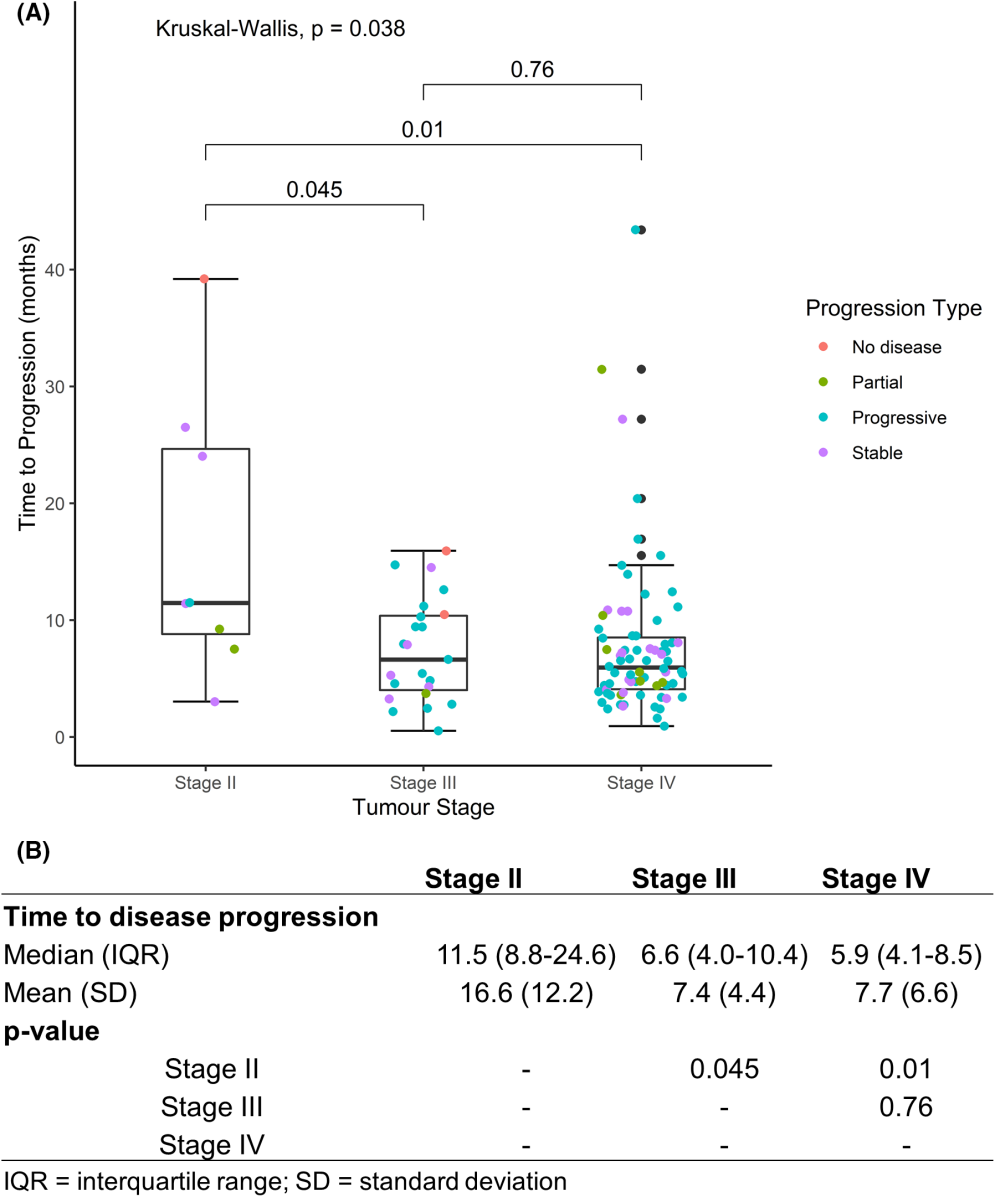
(A) Box plot comparing median time to progression for stage II, III, and IV sub‐cohort; (B) Table describing number of patients in different sub‐groups of stage against disease response. Box Plot representation: Box denotes IQR (25%–75%) range; Horizontal line inside the box denotes median value; Whiskers represents 1.5*IQR; black dot represents an outlier.

The time‐to‐disease progression for patients with different stages of disease was analyzed for 107 patients who received at least three cycles of chemotherapy with post‐treatment assessment scans at TMC. The median duration of progression was 10 months for stage II (*N* = 8) and 5 months for stages III (*N* = 23) and IV (*N* = 76), respectively (*p* = 0.038) (Figure 3). A response to therapy was observed in 7/8 (88%), 8/23 (35%), and 25/76 (33%) patients with stage II, III, and IV diseases, respectively (Table 4).

**TABLE 4:** Disease progression post adjuvant chemotherapy

Stage	*N*	Remission	No remission		
			Stable	Partial	Progressive
Stage II	8	1 (12.5)	4 (50)	2 (25)	1 (25)
Stage III	23	2 (9)	5 (22)	1 (4)	15 (65)
Stage IV	76	0	17 (22)	8 (10)	51 (67)

*Note*: Figures in () represent % of *N* in rows.

## 4 DISCUSSION

The modern management of GBC is a multidisciplinary one. At TMC, patients may initially present to the surgical gastrointestinal‐hepatobiliary, digestive diseases, or medical oncology departments. Depending on the requirements, patients can then be cross‐referred between specialties, with additional referrals to radiation oncology and palliative care. Diagnostic referrals are to radiology and pathology. Given this complexity, these retrospective analyses benefited from the detailed records available in the EMR. Data acquired in structured form allowed the use of natural language processing tools to query and collate data systematically. For the unstructured data, lack of uniformity in documentation was challenging and required considerable manual curation and validation. Thus, while the data presented are verifiable, the usual limitations of retrospective cohort analyses from clinical data sources not designed for research still apply. Data recorded by clinicians did not always contain all the parameters analyzed in this study, so patients with missing data had to be excluded. Where radiology and pathology were assessed elsewhere often the original reports/blocks/films were unavailable. Toxicity data for patients receiving chemotherapy was not available from the records, so we are unable to comment on the severity, though most patients received their blocks of chemotherapy on time. Follow‐up data required contacting families as most patients did not die at TMC. Patients where families could not be contacted to ascertain outcomes were excluded from the survival analysis. Moving forward an objective questionnaire has been designed to collect information prospectively.

Earlier studies have reported a high prevalence of GBC in West Bengal and Kolkata region (~6% of all cancers in the region).^14^, ^15^, ^16^ This accounts for the high numbers of GBC patients seen at TMC Kolkata and we also see a higher incidence in females. A third of the patients in this cohort had type II diabetes mellitus, higher than the reported age adjusted prevalence of this disease (13.5%–17.7%) in India.^17^ Half of the patients were hypertensive, higher than the reported prevalence of 10%–12% in West Bengal.^18^ Both these co‐morbidities have been associated with poor outcomes in GBC and in general in patients with cancer.^19^, ^20^ In this study, gallstones supposedly a precursor of gallbladder adenocarcinoma, were present in 58%. It is quite possible that the actual number may be higher as of the 341 patients who had gallbladder, 161 (47%) were operated prior to coming to TMC. We do not have data on presence of stones/polyps for these patients. This may have contributed to the low prevalence of gallstone and polyp in this retrospective study.

While a simple cholecystectomy may suffice for patients with stage I disease, the curative approach for GBC involves radical cholecystectomy followed by chemotherapy in patients with non‐metastatic disease. Our practice is to attempt radical surgery in patients with unresected/incompletely resected tumors even in those with extensive liver extension as this is associated with better outcomes.^9^ As reported here, around a third of patients with stage II/III disease have had a simple cholecystectomy prior to referral in whom radical surgery was not possible. Outcomes were better in patients who had surgery. The best OS was seen in patients with Stage II who had radical cholecystectomy, though this was not significantly better than those who had simple cholecystectomy. For patients with stage II/III disease where a systematic multimodal approach was achieved showed improvements in outcomes in the last decade at our and other centers,^21^ over those reported previously,^22^, ^23^, ^24^ though in over half of the patients with stage II, disease will recur or progress after therapy. In carefully selected stage IV patients, specially those who remain radiologically stable after neoadjuvant chemotherapy, radical cholecystectomy has been shown to prolong survival. The majority of patients (>80%) in India present with stage III–IV disease with poor performance status where currently a curative approach is not available.

TMC is a tertiary care referral center and most patients come with a provisional diagnosis and usually after having had a surgery. The rate of IGBC in this series (30%) is similar to previously reported (32%),^25^ and this is a worldwide problem.^6^ Pre‐operative imaging and intraoperative findings at the time of laparoscopic cholecystectomy may aid the diagnosis of GBC in patients otherwise diagnosed to have cholecystitis.^26^ Patients suspected to have gallbladder malignancy based on radiological findings, and planned for laparoscopic surgery, should undergo cholecystectomy with *en masse* excision of 3 cm of adjacent liver over the gallbladder fossa. Utmost care should be taken to avoid puncturing the gallbladder and bile spillage. The specimen should be retrieved in a plastic bag and subjected to frozen section. If this shows malignancy, and is more than T1a, then surgery should be converted to an open radical cholecystectomy. In 5.3% of the cases reported here the diagnosis of GBC was missed on initial histopathological examination. For IGBC, radical cholecystectomy needs to be performed within 6–8 weeks of the first surgery,^27^ but the majority of patients at TMC presented later than that, often with metastatic disease. The lower incidence of IGBC previously reported^11^ suggests that patients are presenting at more advanced stages. Nevertheless, the insidious onset with non‐specific symptoms makes an early diagnosis difficult and most patients still present with advanced disease. Given the high incidence of cholelithiasis in Eastern India, a strategy of screening for cholelithiasis and prophylactic cholecystectomy is neither practical or cost‐effective.^28^ Thus, early detection of GBC remains elusive.

Clinical trials in GBC have focused on gemcitabine and platinum‐based adjuvant or neo adjuvant chemotherapy in the broader context of biliary tract cancers (BTC). However, GBC has molecular and clinical characteristics distinctive from other BTC's.^29^, ^30^ The standard chemotherapy at our center is a combination of gemcitabine and cisplatin,^31^ but of late capecitabine has become the drug of choice.^31^, ^32^ Patients who progress on this combination often respond to second‐line therapy with irinotecan/oxaliplatin and fluorouracil.^33^ Limited data suggest neoadjuvant therapy may improve resectability, particularly when oxaliplatin/fluorouracil^34^ is used in comparison to gemcitabine/cisplatin.^35^, ^36^, ^37^, ^38^ At TMC, patients who could not have radical resection but received chemotherapy, had significantly prolonged survival times when compared to patients who were managed symptomatically. While ultimately disease progressed, even patients with stage IV disease were observed to have stable disease over a period of time. This suggests that GBC is chemosensitive but cells rapidly develop resistance to the drugs being used and evaluation of a wider range of available chemotherapeutic agents has merit. This may be particularly beneficial to the majority of patients who currently have non‐resectable disease and may be offered neoadjuvant chemotherapy. Potentially, the development of ex vivo organoid models of GBC may offer pre‐clinical models to evaluate both repurposing of drugs and identifying novel compounds.^39^, ^40^

Our study confirms that GBC continues to be highly prevalent in our region, patients mostly present with advanced disease which is unresectable. Outcomes remain poor and though there is evidence that the tumor may respond to chemotherapy, only a handful of drugs have been evaluated. Given the rarity of this disease in the west, collaborative multicenter interventional clinical trials, particularly adaptively designed umbrella studies, are required in the regions of the world where the disease is more prevalent.

## AUTHOR CONTRIBUTIONS

**Anindita Dutta:** Conceptualization (lead); data curation (lead); formal analysis (lead); funding acquisition (supporting); investigation (lead); methodology (equal); project administration (lead); resources (supporting); software (supporting); supervision (lead); validation (lead); visualization (lead); writing – original draft (lead); writing – review and editing (lead). **Tushar Mungle:** Conceptualization (supporting); data curation (equal); formal analysis (supporting); funding acquisition (supporting); investigation (supporting); methodology (equal); project administration (supporting); resources (supporting); software (lead); supervision (supporting); validation (equal); visualization (equal); writing – original draft (supporting); writing – review and editing (supporting). **Nandita Chowdhury:** Data curation (lead); formal analysis (supporting); software (supporting); visualization (supporting); writing – review and editing (supporting). **Pritha Banerjee:** Data curation (lead); formal analysis (supporting); software (supporting); visualization (supporting); writing – review and editing (supporting). **Anisha Gehani:** Investigation (lead). **Saugata Sen:** Investigation (lead); writing – review and editing (lead). **Mohandas Mallath:** Investigation (lead). **Paromita Roy:** Investigation (lead). **Shekhar Krishnan:** Conceptualization (supporting); funding acquisition (supporting); methodology (supporting); writing – original draft (supporting); writing – review and editing (supporting). **Sandip Ganguly:** Investigation (lead); writing – review and editing (supporting). **Sudeep Banerjee:** Investigation (lead); writing – review and editing (supporting). **Manas Roy:** Investigation (lead); writing – review and editing (supporting). **Vaskar Saha**: Conceptulization (equal); formal analysis (supporting); funding acquisition (lead); resources (equal); supervision (lead); validation (supporting); visualization (supporting); writing ‐ original draft (lead); writing ‐ review and editing (lead).

## ETHICS APPROVAL STATEMENT

Ethical approval for this research study was waived by Tata Medical Center's Institutional Review Board (EC/WV/TMC/22/22).

## Supporting information


Figure S1

Table S1
Table S2
Table S3
Table S4

## Data Availability

Anonymized data are available from the corresponding author upon request.
