# Supplementary figures and images for: Characteristics and outcomes of gallbladder cancer patients at the Tata Medical Center, Kolkata 2017–2019
Source: Cancer Med. 2023 Feb 13;12(8):9293–302. doi: 10.1002/cam4.5677 (PMC10166897; doi:10.1002/cam4.5677)

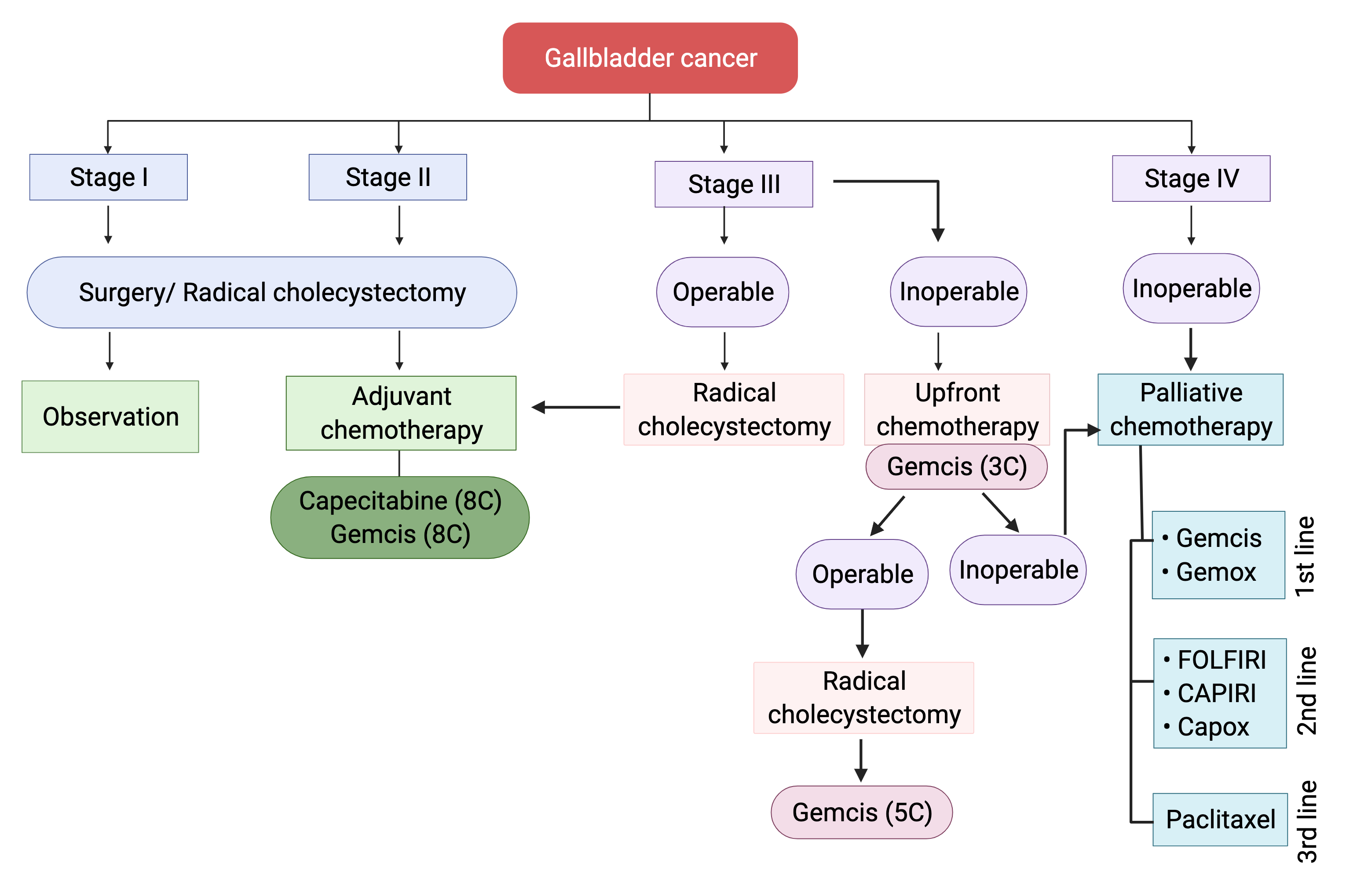

Supplement: Supplementary file 1 — Figure S1 [file CAM4-12-9293-s002.png]
